# Detecting and confirming residual hotspots of lymphatic filariasis transmission in American Samoa 8 years after stopping mass drug administration

**DOI:** 10.1371/journal.pntd.0005914

**Published:** 2017-09-18

**Authors:** Colleen L. Lau, Sarah Sheridan, Stephanie Ryan, Maureen Roineau, Athena Andreosso, Saipale Fuimaono, Joseph Tufa, Patricia M. Graves

**Affiliations:** 1 Department of Global Health, Research School of Population Health, The Australian National University, Canberra, Australia; 2 Children’s Health and Environment Program, Child Health Research Centre, The University of Queensland, Brisbane, Australia; 3 Australian Institute of Tropical Health and Medicine and College of Public Health, Medical and Veterinary Sciences, James Cook University, Cairns, Australia; 4 Department of Public Health, American Samoa Department of Health, Pago Pago, American Samoa; Imperial College London, UNITED KINGDOM

## Abstract

The Global Programme to Eliminate Lymphatic Filariasis (LF) aims to eliminate the disease as a public health problem by 2020 by conducting mass drug administration (MDA) and controlling morbidity. Once elimination targets have been reached, surveillance is critical for ensuring that programmatic gains are sustained, and challenges include timely identification of residual areas of transmission. WHO guidelines encourage cost-efficient surveillance, such as integration with other population-based surveys. In American Samoa, where LF is caused by *Wuchereria bancrofti*, and *Aedes polynesiensis* is the main vector, the LF elimination program has made significant progress. Seven rounds of MDA (albendazole and diethycarbamazine) were completed from 2000 to 2006, and Transmission Assessment Surveys were passed in 2010/2011 and 2015. However, a seroprevalence study using an adult serum bank collected in 2010 detected two potential residual foci of transmission, with Og4C3 antigen (Ag) prevalence of 30.8% and 15.6%. We conducted a follow up study in 2014 to verify if transmission was truly occurring by comparing seroprevalence between residents of suspected hotspots and residents of other villages. In adults from non-hotspot villages (N = 602), seroprevalence of Ag (ICT or Og4C3), Bm14 antibody (Ab) and Wb123 Ab were 1.2% (95% CI 0.6–2.6%), 9.6% (95% CI 7.5%-12.3%), and 10.5% (95% CI 7.6–14.3%), respectively. Comparatively, adult residents of Fagali’i (N = 38) had significantly higher seroprevalence of Ag (26.9%, 95% CI 17.3–39.4%), Bm14 Ab (43.4%, 95% CI 32.4–55.0%), and Wb123 Ab 55.2% (95% CI 39.6–69.8%). Adult residents of Ili’ili/Vaitogi/Futiga (N = 113) also had higher prevalence of Ag and Ab, but differences were not statistically significant. The presence of transmission was demonstrated by 1.1% Ag prevalence (95% CI 0.2% to 3.1%) in 283 children aged 7–13 years who lived in one of the suspected hotspots; and microfilaraemia in four individuals, all of whom lived in the suspected hotspots, including a 9 year old child. Our results provide field evidence that integrating LF surveillance with other surveys is effective and feasible for identifying potential hotspots, and conducting surveillance at worksites provides an efficient method of sampling large populations of adults.

## Author summary

Lymphatic filariasis (LF) is caused by infection with filarial worms that are transmitted by mosquito bites. The Global Programme to Eliminate Lymphatic Filariasis aims to eliminate the disease as a public health problem by 2020. Once elimination targets have been reached, cost-effective surveillance strategies are required to ensure that any areas of ongoing transmission or resurgence are quickly identified and managed. Potential options include the integration of LF surveillance with other public health activities. In American Samoa, blood samples collected in 2010 for a research project on a different disease (leptospirosis) were used to test for evidence of LF infection, and the study found two possible areas of ongoing transmission. We conducted a follow up study in 2014 to verify whether LF infection was truly occurring in these two areas, and found that infection rates in both areas were significantly higher compared to other parts of American Samoa. Our results therefore provide field evidence that integrating LF surveillance activities with other population-based surveys are potentially effective and feasible, and provide a cost-effective method for identifying residual areas of transmission.

## Introduction

Lymphatic filariasis (LF) is a mosquito-borne parasitic infection caused by *Wuchereria* or *Brugia* species of helminths. Mosquito vectors vary between countries and regions, and include *Aedes*, *Anopheles*, *Culex* and *Mansonia* species. Globally, it is estimated that 68 million people are affected, comprising approximately 36 million microfilaraemic persons and 36 million with disabling complications such as severe lymphedema, including elephantiasis and scrotal hydroceles [1]. The Global Programme to Eliminate LF (GPELF) was launched by the World Health Organization in 2000, with the aim of eliminating the disease as a public health problem by 2020. The program consists of two components: i) to interrupt transmission through mass drug administration (MDA) and ii) to control morbidity and disability of affected populations [2]. As part of GPELF, the Pacific Programme to Eliminate LF (PacELF) was formed in 1999 to focus on 22 Pacific Island Countries and Territories (PICTs). PacELF focused on coordinating the elimination efforts in the PICTs, which include >3000 islands and 8.6 million people [3,4].

Since 2000, the GPELF has made impressive progress globally, with a total of 6.2 billion treatments delivered to >820 million people [2]. Once elimination targets have been reached, effective monitoring and surveillance are critical for ensuring that programmatic gains are sustained in the long-term. The World Health Organization and GPELF have identified some key operational challenges in post-MDA surveillance, including i) timely identification of residual areas of high-prevalence and/or resurgence, ii) strategies for managing these high risk areas, and iii) development of cost-effective surveillance strategies [5,6].

In American Samoa, a group of remote islands in the South Pacific, LF is caused by the diurnally sub-periodic *W*. *bancrofti*, and the main mosquito vector responsible for transmission is the highly efficient day-biting *Ae*. *polynesiensis*. Other vectors include *Ae*. *samoanus* (night-biting), *Ae*. *tutuilae* (night-biting), and *Ae*. *upolensis* (day-biting) [7–10]. Two rounds of MDA in 1963 and 1965 reduced microfilaria (Mf) prevalence from ~20% to <2% [4,11,12]. Unfortunately, transmission was not successfully interrupted, and antigen prevalence measured by rapid immunochromatographic test (ICT) had risen to 16.5% (N = 3018) when the PacELF baseline survey was conducted in 1999. Since then, American Samoa has made significant progress towards LF elimination. After seven rounds of MDA from 2000–2006, antigen prevalence dropped to 2.3% (N = 1881) in a community cluster survey in 2007 [13–15]. However, the results of the 2007 survey did not meet PacELF’s criteria for stopping MDA (<1% antigenaemia, upper 95% CI <2%), and an additional round of MDA was recommended, but no further effective rounds of MDA were successfully completed after this time [15].

The WHO currently recommends post-MDA surveillance using transmission assessment surveys (TAS), which use critical cut-off values of numbers of antigen-positive children aged 6–7 years to determine whether transmission has been interrupted in defined evaluation units [16]. In areas where *W*. *bancrofti* is endemic and *Aedes* is the principal vector, the target threshold for post-MDA transmission assessment surveys (TAS) is <1% antigenaemia [16]. American Samoa passed TAS-1 in 2011–2012 [17] and TAS-2 in 2015 [18], but recent human seroprevalence studies and molecular xenomonitoring studies of mosquitoes identified epidemiological and entomological evidence of ongoing LF transmission [10,19,20].

As prevalence drops to very low levels in the end stages of elimination programs, not only will it become more challenging to detect any residual hotspots of ongoing transmission, but funding and resources for programmatic activities will also generally be reduced. The current WHO guidelines therefore encourage cost-efficient methods for post-MDA surveillance, including the integration of LF surveillance activities with other population-based surveys, and opportunistic screening of large groups (e.g. military recruits, hospital patients, and blood donors) for microfilaraemia, antigenaemia, or antibodies [16]. For example, in Togo [21] and Vanuatu [22], nationwide LF surveillance has been successfully conducted by screening blood smears collected for malaria diagnosis. However, there is currently little evidence about the effectiveness of these strategies for identifying infected persons in the post-MDA setting.

Lau et al previously reported a study of the seroprevalence and spatial epidemiology of LF in American Samoa after successful MDA [19]. The study used a serum bank collected from adults (aged ≥18 years) for a leptospirosis study in 2010 [23], four years after the last effective round of MDA. The study found epidemiological evidence of possible residual foci of Og4C3 Ag-positive people in two localised areas, with an average cluster size of ~1.5 km. One cluster was found in the very small village of Fagali’i, where the seroprevalence of Og4C3 Ag was 30.8% (95% CI 9.1–61.4%). Another cluster spanned the contiguous villages of Ili’ili, Vaitogi, and Futiga, where overall Og4C3 Ag prevalence was 15.6% (95% CI 5.3–32.8%) (data derived from [19]). However, the findings were not definitive because information on microfilaraemia was not available; the study was conducted using a pre-existing serum bank and the findings were based entirely on serological markers. In this paper, we report results of a follow up study in 2014 to confirm whether there was indeed ongoing transmission and/or higher infection rates in the two suspected ‘hotspot areas’ identified from the previous work. If ongoing transmission was truly occurring in these two suspected hotspot areas, our findings would provide field evidence to support WHO’s recommendations for integrating LF surveillance activities with other population-based surveys, including those that only include adults [16].

## Materials and methods

### Ethics statement

Ethics approvals were granted by the American Samoa Institutional Review Board, and the Human Research Ethics Committees at James Cook University (H5519) and The University of Queensland (2014000409). The study was conducted in collaboration with the American Samoa Department of Health, and official permission for village visits was sought from the Department of Samoan Affairs and village chiefs and/or mayors. Verbal and written information were provided to all participants (or their parent or guardian) in Samoan or English according to the participant’s preference. Signed informed consent forms were obtained from all participants, or their parent or guardian if under 18 years of age.

### Study location and setting

American Samoa is a United States Territory in the South Pacific, consisting of a group of small tropical islands with a total population of 55,519 living in ~70 villages (average population ~800 per village) at the 2010 census [24]. Over 90% of the population live on the main island of Tutuila, and the remainder on the adjacent island of Aunu’u and the remote Manu’a group of islands. American Samoa has a tropical climate and is one of the wettest inhabited places in the world, with islands that include mountains, valleys, tropical rainforests, wetlands, fringing reefs, and lagoons.

### Sampling design

Field data were collected from American Samoa in 2014 from the following groups of participants:

1. ***Adult workers*** (aged ≥15 years) recruited from:
  - A Department of Health clinic where workers attend for fitness-for-work medical examinations
  - Starkist tuna cannery (the largest private employer in the territory)
2. ***Community members*** (aged ≥2 years) from the two suspected hotspot areas identified by the previous study [19]:
  - The contiguous villages of Ili’ili, Vaitogi and Futiga, with a combined population of 5,877 [24]
  - Fagali’i, a very small village on the far western end of Tutuila, with a population of 247 [24]
3. ***School children*** (Grades 3 to 8, aged 7 to 13 years) from the large elementary school in Ili’ili. This school was included in the study because it was the only school where ICT-positive children were identified in the 2011/2012 TAS-1 [17], and also located in Ili’ili, within one of the suspected hotspot areas. The school survey provided a convenient and cost-effective method of sampling a large number of children who lived in Ili’ili, Vaitogi, and Futiga. Grades 1 and 2 children were not included in our 2014 study because they were to be tested in the 2015 TAS-2 a few months after our survey. There were no other elementary schools in Ili’ili, and no schools located in Fagali’i, the other suspected hotspot area.

Adult workers and village residents were recruited by convenience sampling because probability-based sampling was not logistically possible with the available budget and resources. The field team was stationed at the weekly Department of Health clinic from May to December 2014 (~4 hours per visit), and invited all clinic attendees to participate. Visits to the tuna cannery, villages, and school were conducted over a 3-week period in October and November 2014. The team visited the tuna cannery on four occasions (~4 hours per visit), and all employees on duty were invited to participate. For visits to the suspected hotspot areas, permissions were sought from village chiefs and mayors, who informed residents of the team’s pre-arranged visits. During the three village visits to Ili’ili/Vaitogi/Futiga, and one village visit to Fagali’i (~4 hours per visit), the field team was positioned in a prominent and central part of the community, and all residents were invited to participate. All children in Grades 3 to 7 who attended the elementary school in Ili’ili were invited to participate; information sheets and consent forms were distributed to parents and guardians about one week beforehand, and all children who returned valid consent forms were tested.

### Collection of samples and data

The following samples and data were collected from each participant:

1. Blood samples, collected by trained phlebotomists under sterile conditions:
  - Venous samples (~5mL) were collected from adult workers and community members
  - Finger prick samples were collected from school children into heparinised capillary tubes (100 μL) and whole blood collected onto TropBio filter papers (10 μL per ear, max 6 ears per participant). The filter papers were allowed to dry thoroughly and then placed in individual sealed plastic bags.
2. Questionnaire data, using standard questionnaires administered by bilingual research assistants. Information on demographics, country of birth or years lived in American Samoa, and current village of residence were collected from all participants. Questions for community members and adult workers also included occupation, years lived in American Samoa, history of taking MDA, and previous diagnosis of lymphatic filariasis (by a doctor or other health care worker).
3. Household location data, using detailed village maps generated from high-resolution geographic information system (GIS) data (except for school children where only the village of residence was recorded).

Data on population demographics were sourced from the 2014 American Samoa Statistical Year Book [24], and high-resolution GIS data were provided by the American Samoa GIS User Group [25].

### Serological analysis and microscopic examination for microfilaria

Venous or fingerprick blood samples were tested for filarial antigen immediately after collection using the Alere BinaxNOW Filariasis immunochromatographic test (ICT). If an ICT was positive, the result was confirmed by repeating the test, and two Mf slides were prepared, each with 60 μL of blood in 3 lines of 20 μL each per slide. Once thoroughly dried, slides were dehaemoglobinized, fixed with methanol and stained with 2% Giemsa stain for 50 minutes according to WHO guidelines [16] and examined at 100x magnification. Mf densities in 60 μL were determined by counting all Mf on each slide. Each set of Mf slides were read blindly by two or three experienced parasitologists, one at James Cook University Cairns (PG) and the other(s) at the LBJ Tropical Medical Centre in American Samoa and/or at James Cook University in Townsville, Australia. Counts were converted to Mf/mL and the final Mf density recorded was the average of the counts reported by two or three parasitologists.

Venous blood samples were allowed to clot before centrifuging, and serum were aliquoted and stored at -20 degrees Celsius in American Samoa. Frozen sera and dried blood spots (DBS) were shipped to Australia for serological analysis at James Cook University, Cairns, Australia. All samples were tested for Og4C3 Ag using the TropBio Og4C3 Filariasis Antigen ELISA test (Cellabs Pty. Ltd., New South Wales, Australia) using dilutions for serum and DBS recommended by the manufacturer. Bm14 Ab was measured using ELISA tests (CDC in house version) as previously described [19]. For Wb123 Ab, all the village samples and 109 of the adult worker samples were tested with in-house Wb123 ELISA as previously described [19]. The remaining 552 adult worker samples and 178 of the school children samples were tested using the InBios Wb123 ELISA [26]. Due to insufficient volumes of blood available, 149 children did not have Wb123 ELISA done by either method. All ELISAs used standard curves with kit provided standards (Og4C3 Ag) or known strong positives (Bm14 Ab and Wb123 Ab). Cutoffs for positivity were aligned between the two Wb123 Ab methods.

### Treatment of ICT-positive individuals

All ICT-positive individuals were treated with albendazole (400mg) and diethyl-carbamazine (DEC) (6mg/kg) according to WHO recommended dosages, and all treatments were provided free of charge. Children were treated with informed consent from and in the presence of at least one parent or guardian.

### Statistical analysis

The outcome measures used were positive results for ICT, Og4C3 Ag >32 units (weak positive), Og4C3 Ag >128 units (positive), ‘antigen’ (ICT and/or Og4C3 Ag >32 units), Bm14 Ab, Wb123 Ab, and Mf. The positivity levels for Og4C3 Ag were chosen based on product information provided by Cellabs, and a previous study in American Samoa [19].

To determine whether residents of suspected hotspot areas had higher infection rates than residents of other villages, seroprevalence of adult workers who resided in other villages were used as the reference group to provide estimates of infection rates in the general population.

Simple proportions were compared using Chi-squared tests or Fisher exact tests, and binomial exact 95% confidence intervals. Point estimates of antigen and antibody prevalence were calculated for residents of each of the suspected hotspot areas and adult workers who lived in other villages. Prevalence estimates were standardised for age using American Samoa’s age distribution data from the 2014 Statistical Yearbook [24], and 95% confidence intervals calculated using the ‘stdize’ option in the ‘proportion’ command in Stata 14, with ‘stdweights’ as the proportion of the population in each age group. Statistical associations between place of residence and presence of serological markers were quantified using univariable logistic regression (weighted for age distribution). Because adult workers were used as the reference population, village residents aged <15 years were excluded from the logistic regression analyses.

Data were managed using Microsoft Excel (v14, 2011) and Qualtrics (Qualtrics, Provo, UT), an electronic platform for collecting and managing data. Stata 14 (StataCorp, College Station, TX) was used for data analyses, and *p* values of <0.05 were considered statistically significant.

## Results

### Study population

The study included a total of 1,132 participants, comprising 172 employment clinic attendees, 498 tuna cannery workers, 125 community members from the two suspected hotspot areas, and 337 school children who attended an elementary school in Ili’ili. The school children represented 61.6% of the total 547 students enrolled in Grades 3 to 8 at the school. Of the 337 children, 283 (84.0%) were residents of Ili’ili/Vaitogi/Futiga, one of the suspected hotspot areas. Children who attended the school but resided in other villages were also tested, but not included in the statistical analyses for residents of suspected hotspot areas because most lived near the school and were therefore not representative of the general population of children in American Samoa.

The participants were classified into three groups for statistical analyses:

1. Residents of Ili’ili/Vaitogi/Futiga (N = 418, aged ≥2 years), including:
  - 66 adult workers who resided in these villages
  - 69 community members recruited from field visits to these villages
  - 283 school children who resided in these villages
2. Residents of Fagali’i (N = 58, aged ≥2 years), including:
  - 2 adult workers who lived in this village
  - 56 community members recruited from a field visit to the village
3. Adult workers who did not live in either of the suspected hotspot areas (N = 602, aged ≥15 years), including:
  - 151 employment clinic attendees
  - 451 cannery workers

**Fig 1:**
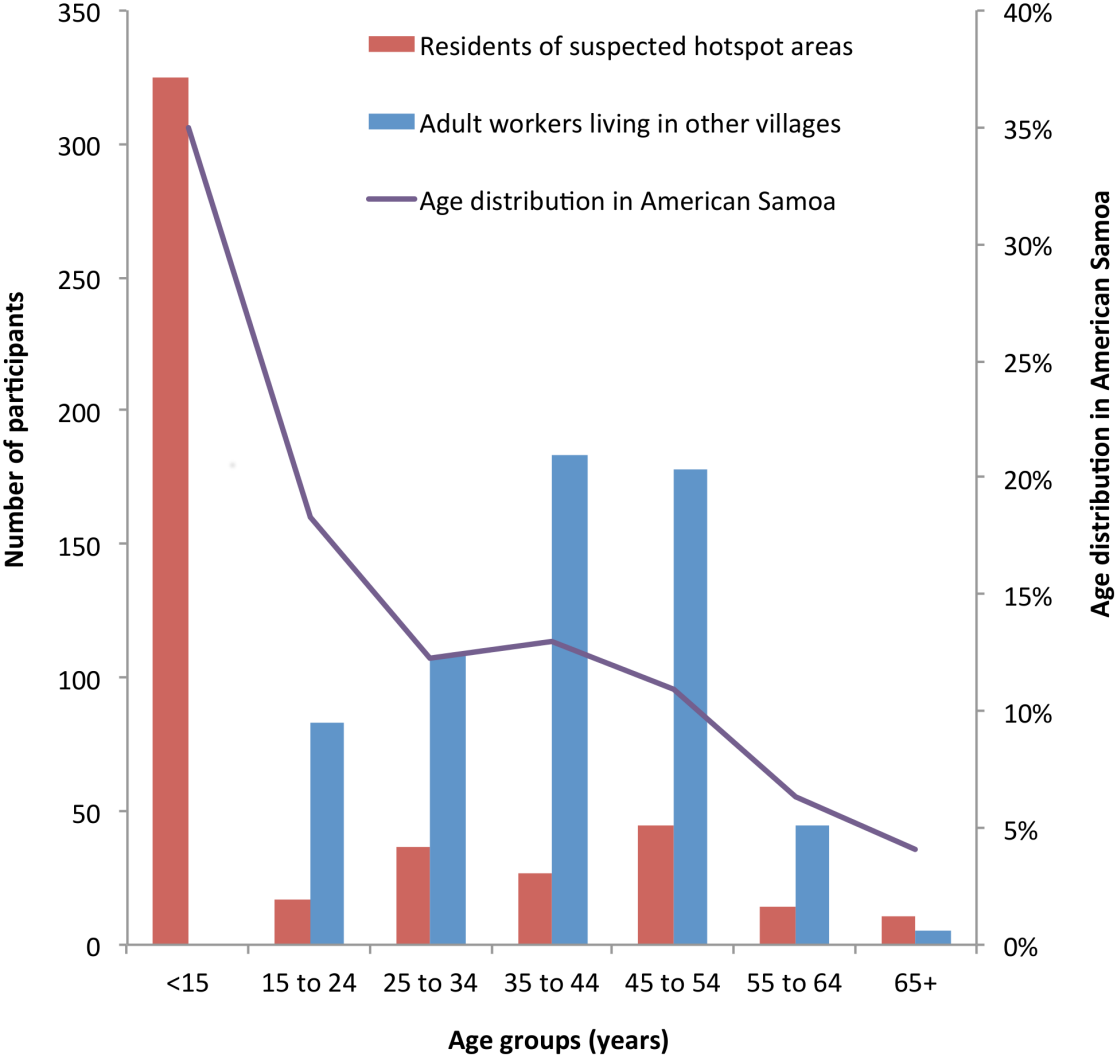
Age distributions of residents of suspected hotspot areas, adult workers living in other villages, and the general population in American Samoa.

Fig 1 shows the age distributions of residents of suspected hotspots, adult workers from other villages, and American Samoa’s general population. A summary of the representativeness of each study population is shown in Table 1.

**Table 1:** Summary of the representativeness of study participants.

Group	Total population	Number tested	% of population tested	Female:Male ratio	% (95% CI) of adults who have lived in American Samoa since 2000^a^
Residents of Ili’ili, Vaitogi, and Futiga	5,877	418	7.1%	1.33	75.7% (66.6–83.3%)
Residents of Fagali’i	247	58	23.5%	1.38	75.7% (58.8–88.2%)
Adult workers living in other villages	37,046^b^	602	1.6%	1.56	70.7% (66.8–74.3%)

^a^ Indicates persons who have lived in American Samoa since the PacELF program commenced in 2000

^b^ Estimated total population of American Samoans aged ≥15 years, based on Statistical Yearbook 2014 [24]

**Fig 2:**
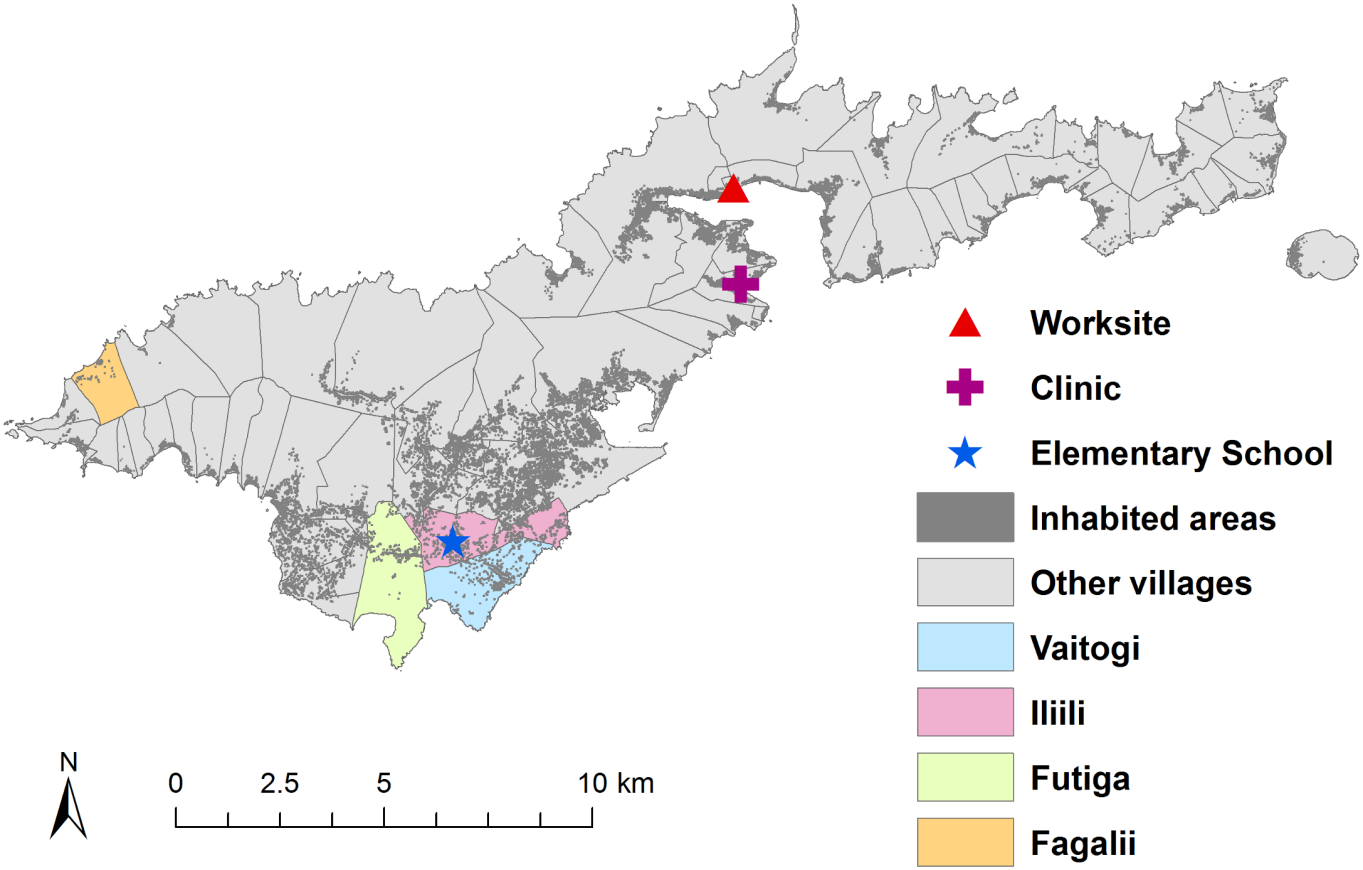
Population distribution, location of the two suspected hotspot areas, and the elementary school, clinic, and worksite surveyed in the study. GIS data were provided by the American Samoa GIS user group [22].

**Fig 3:**
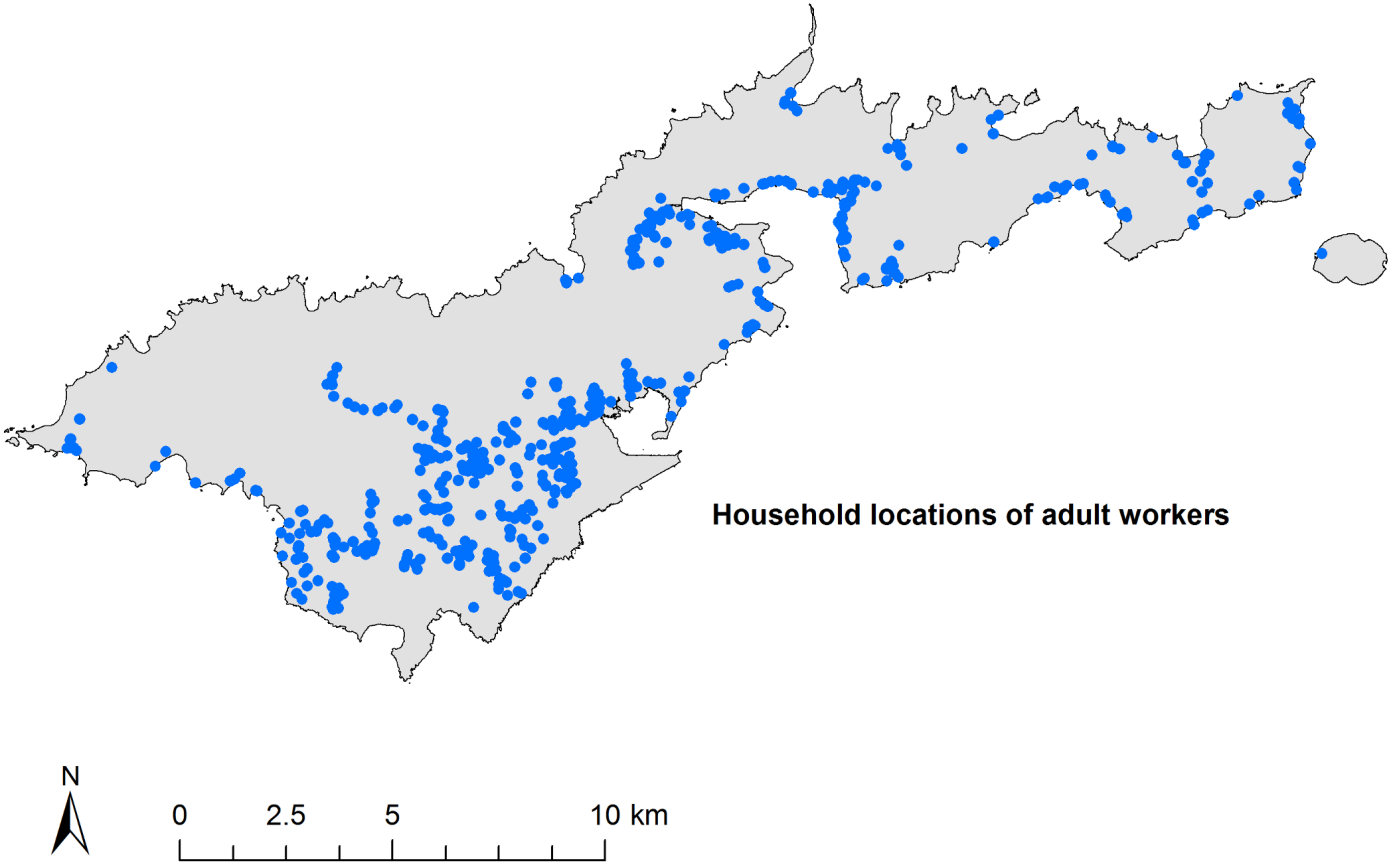
Residential locations of adult workers recruited from pre-employment clinic and tuna cannery (blue circles). GIS data were provided by the American Samoa GIS user group [22].

Fig 2 shows the distribution of the general population on Tutuila, the locations of the suspected hotspot areas included in this study, the elementary school in Ili’ili where children were tested, and the clinic and tuna cannery where adult workers were tested. Fig 3 shows the residential locations of all adult workers who participated in this study. Although the adult workers were sampled at one clinic and one work site, they resided across 51 villages on the main island of Tutuila and the adjacent island of Aunu’u. Considering that convenience sampling was used in this study, the adult workers provided a reasonably representative sample of the general adult population in terms of place of residence.

Overall, 72.3% (95% CI 61.4% to 81.6%) of adult community members and 67.6% (95% CI 63.8% to 71.3%) of adult workers reported taking MDA in the past, either in American Samoa and/or elsewhere, while 3.6% (95% CI 0.8% to 10.2%) of adult community members and 1.3% (95% CI 0.5% to 2.5%) of adult workers reported that a doctor or other health worker had previously diagnosed them with LF. There were no statistically significant differences in MDA participation or LF diagnosis between adult community members and adult workers, suggesting that convenience sampling did not introduce any significant participation biases towards either group being more likely to have LF infection.

### Prevalence of antigens and microfilaraemia

A total of 29 antigen-positive individuals were identified from ICT and/or Og4C3 Ag (>32 units) tests, with a female to male ratio of 1.07 and age range of 9 to 73 years. A summary of antigen-positive results for each group is shown in Table 2.

**Table 2:** Summary of antigen results for residents of suspected hotspot areas and adult workers living in other villages.

Group	Number tested	Positive ICT	Positive Og4C3 Ag (>32 units)	Positive Og4C3 Ag (>128 units)	Positive antigen (ICT and/or Og4C3 >32 units)	% of population with positive antigen (95% CI)
Residents of Ili’ili, Vaitogi, and Futiga	418	7	6	4	10	2.4 (1.2–4.4)
Residents of Fagali’i	58	8	9	8	10	17.2 (8.6–29.4)
Adult workers living in other villages	602	8	7	4	9	1.5 (0.7–2.8)
Total	1078	23	22	16	29	2.7 (1.8–3.8)

Of the 337 school children tested, three of the 283 residents of Ili’ili/Vaitogi/Futiga were ICT-positive (1.1%, 95% CI 0.2% to 3.1%), compared to none of the 54 who were residents of other villages (0%, one-sided 97.5% CI 0% to 6.6%).

**Fig 4:**
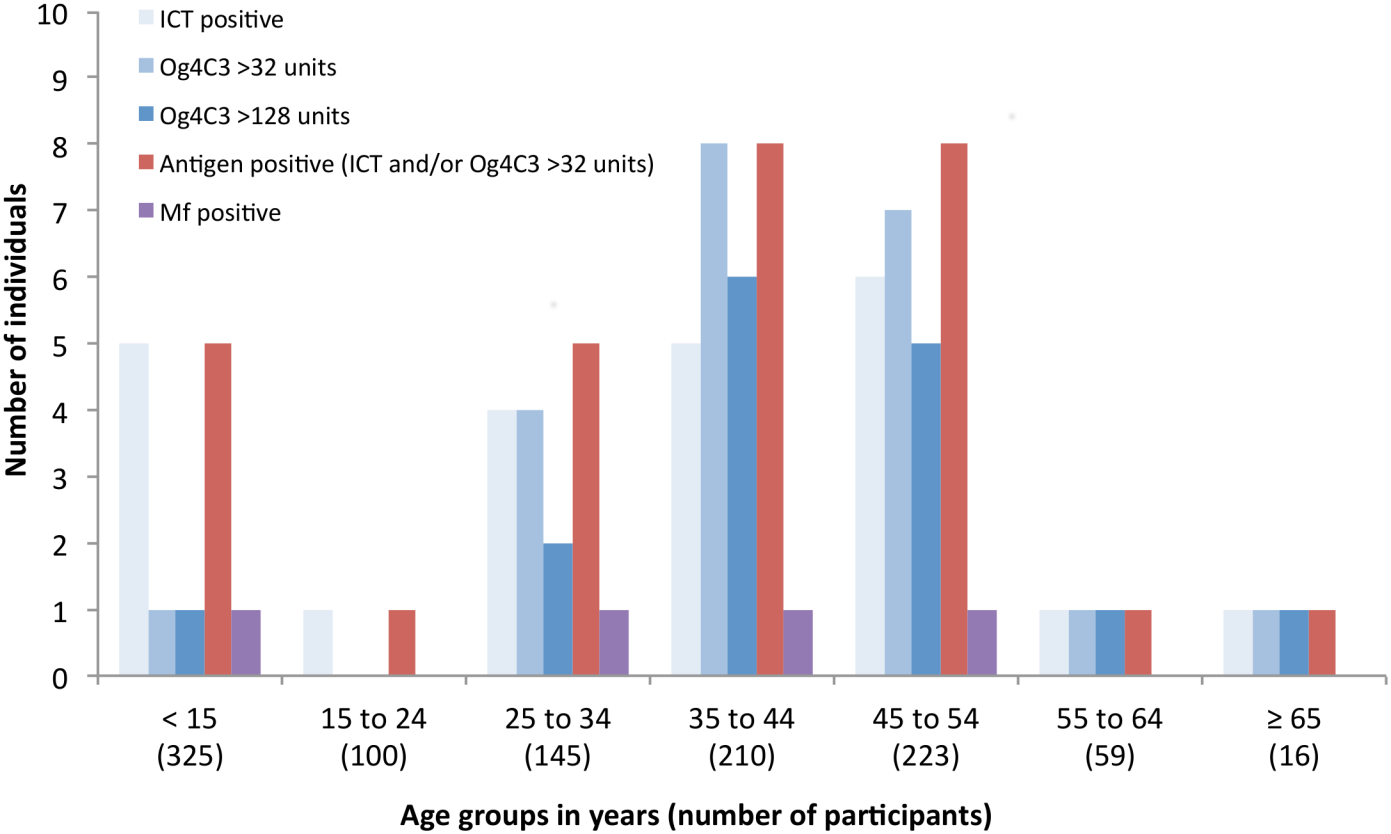
Age distribution of antigen-positive and microfilaraemic individuals (from all survey locations).

Four microfilaraemic individuals were identified out of 20 available slides examined, with Mf densities of 8, 433, 2667, and 3267 Mf/mL. These counts were the average of two or three blind readings of the slides by different parasitologists. The Mf-positive persons were aged 9, 29, 35, and 46 years, with a female:male ratio of 1. All microfilaraemic individuals lived in the hotspot areas; three in Fagali’i and one in Vaitogi. The age distributions of antigen-positive and Mf-positive individuals are shown in Fig 4. Of the 15 ICT-positive people who resided in suspected hotspot areas, Mf results were available for 14, of which 4 (28.6%) were Mf-positive. Of the 8 ICT-positive people who resided in other villages, Mf results were available for 6, of which none were Mf-positive. The difference in proportion of Mf-positive results was not statistically significant, but sample sizes were small.

The number of years lived in American Samoa was not significantly associated with seroprevalence for antigen or antibodies. Antigen prevalence was 3.2% in those who had lived in American Samoa since 2000 (when MDA started), 2.5% in those who arrived after 2006 (when the last effective round of MDA was conducted), and 3.3% in those who arrived between 2000–2006 (Chi-squared test, *p* = 0.89). There were no significant differences in seroprevalence between the three groups for Bm14 Ab (Chi-squared test, *p* = 0.25) or Wb123 Ab (Chi-squared test, *p* = 0.65).

### Age-adjusted seroprevalence of antigen and antibodies

**Fig 5:**
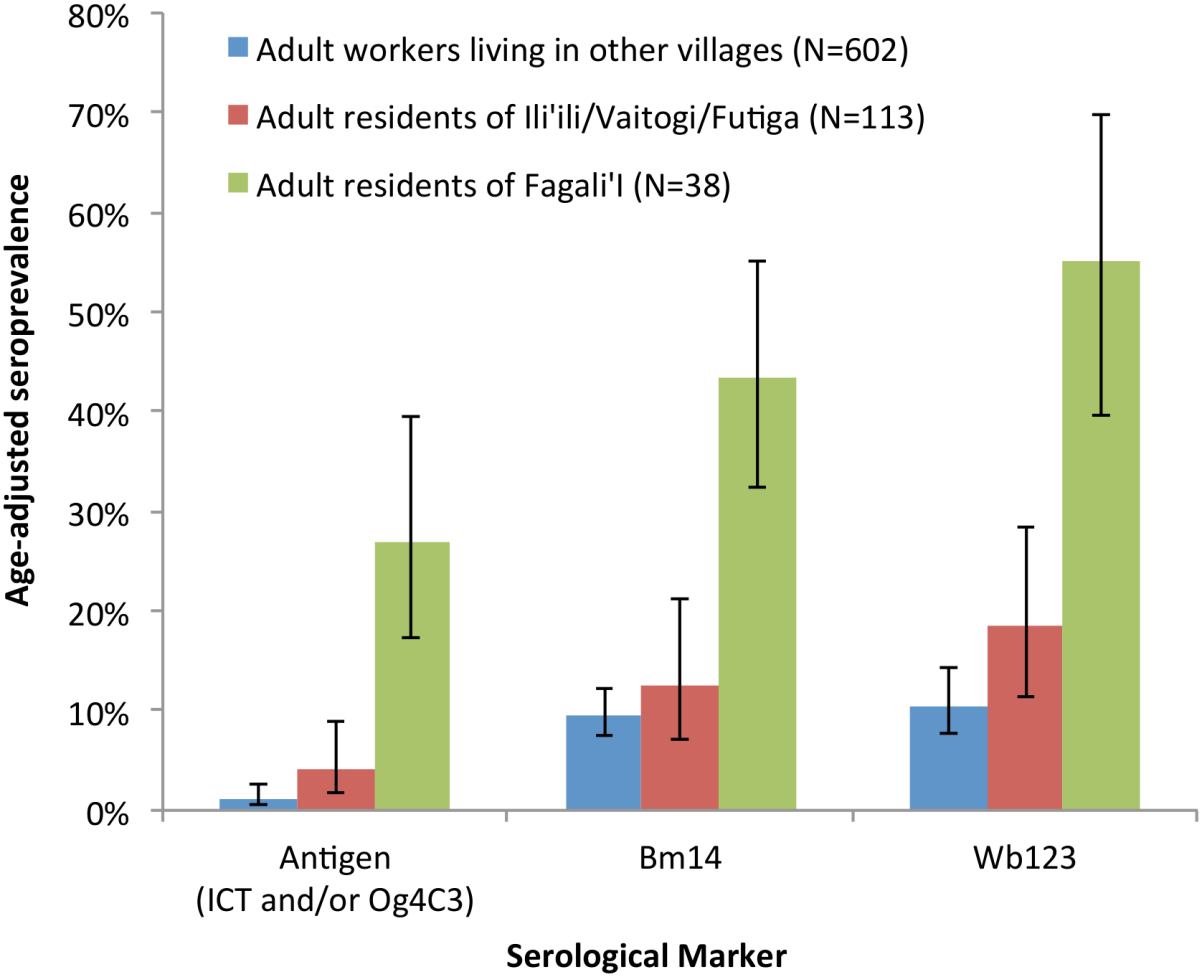
Age-adjusted seroprevalence of antigen (ICT and/or Og4C3 Ag >32 units), Bm14 Ab, and Wb123 Ab in adult residents of suspected hotspot areas and adult workers who lived in other villages.

Estimates of the seroprevalence of LF antigen (positive ICT and/or Og4C3 Ag >32 units), Bm14 Ab, and Wb123 Ab were calculated, and adjusted for age based on the population age distribution reported in the 2010 census [24]. Comparisons of the age-adjusted seroprevalence for adult residents (aged ≥15 years) of each suspected hotspot area and adult workers who resided in other villages are summarised in Fig 5.

In adults who lived outside of hotspot villages, age-adjusted seroprevalence of LF antigen, Bm14 Ab, and Wb123 Ab were 1.2% (95% CI 0.6–2.6%), 9.6% (95% CI 7.5%-12.3%), and 10.5% (95% CI 7.6–14.3%) respectively. Comparatively, adult residents of Fagali’i had significantly higher seroprevalence of antigen (26.9%, 95% CI 17.3–39.4%), Bm14 Ab (43.4%, 95% CI 32.4–55.0%), and Wb123 Ab 55.2% (95% CI 39.6–69.8%) than adults living in other areas. Adult residents of Ili’ili/Vaitogi/Futiga also had higher seroprevalence of antigen (4.0%, 95% CI 1.8–8.8%), Bm14 Ab (12.5%, 95% CI 7.1–21.1%) and Wb123 Ab 18.5% (95% CI 11.4–28.5%) than adults living in other areas, but the differences were not statistically significant.

**Fig 6:**
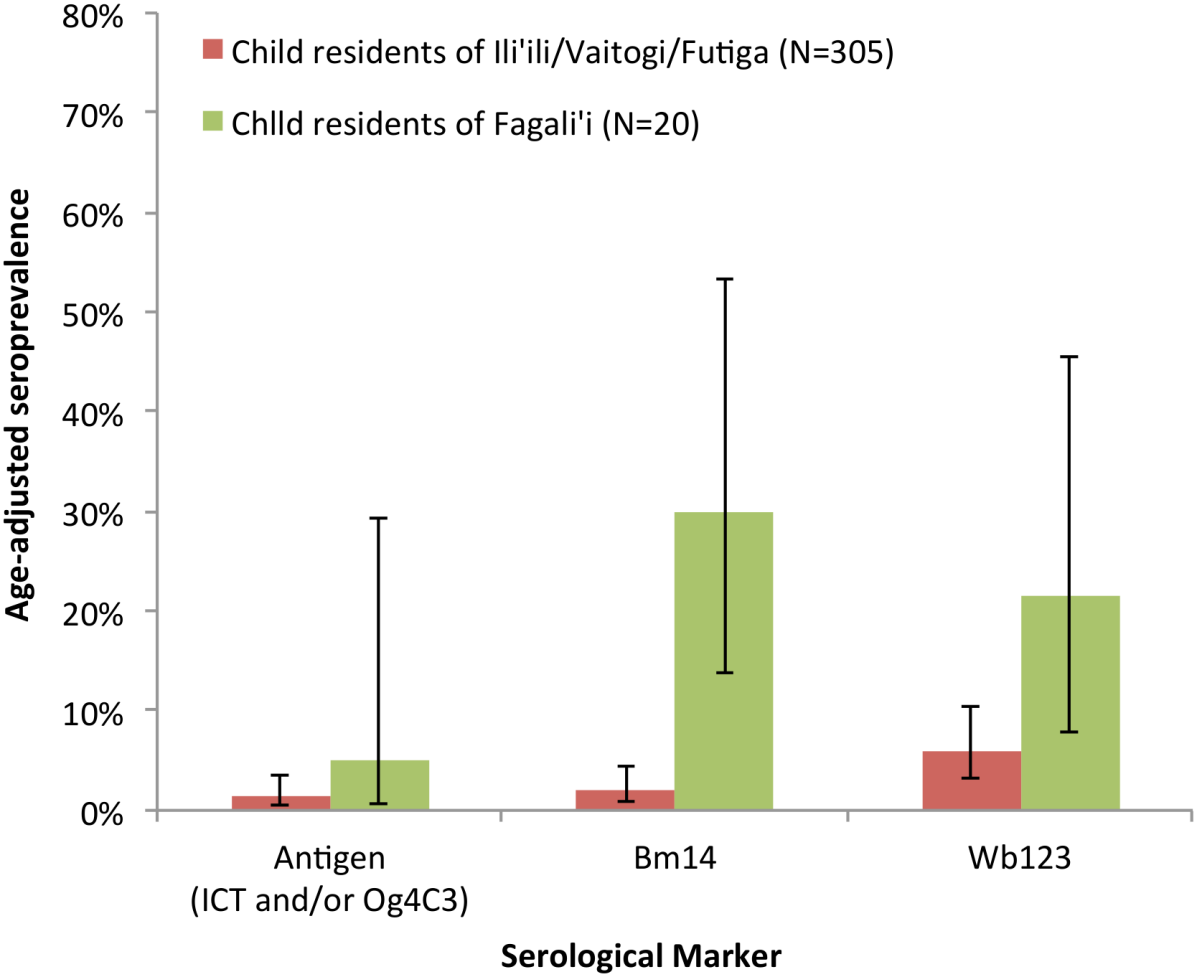
Age-adjusted seroprevalence of antigen (ICT and/or Og4C3 Ag >32 units), Bm14 Ab, and Wb123 Ab in child residents (aged 2–14 years) of suspected hotspot areas.

Comparisons of the age-adjusted seroprevalence of antigen and antibodies in children (aged 2 to 14 years) from each of the suspected hotspot areas are shown in Fig 6. Age-adjusted seroprevalence of antigen and antibodies were higher in child residents of Fagali’i compared to those who lived in Ili’ili/Vaitogi/Futiga: 5.0% (95% CI 0.7–29.5%) versus 1.3% (95% CI 0.5–3.5%) for antigen; 30.0% (95% CI 13.8%-53.4%) versus 2.0% (95% CI 0.9–4.4%) for Bm14 Ab; and 21.5% (95% CI 7.9–45.5%) versus 5.8% (95% CI 3.1–10.5%) for Wb123 Ab. The study did not collect data from children in other villages that were sufficiently representative for meaningful comparison with the results from suspected hotspots. It should be noted that for some children, there were insufficient blood samples for all serological tests to be conducted. Wb123 Ab results were only available for 173 of the 305 (56.7%) children who lived in Ili’ili/Vaitogi/Futiga, and 19 of the 20 (95%) children who lived in Fagali’i.

### Association between presence of serological markers and place of residence in adults

Odds ratios of the presence of antigens and antibodies in residents of suspected hotspot areas were calculated using univariate logistic regression (weighted for age distribution), using adult workers living in other villages as the reference group. Only participants aged ≥15 years were included in this analysis because the reference group did not include any participants aged <15 years. Table 3 shows that residents of both suspected hotspots were significantly more likely to be seropositive. Residents of Fagali’i had significantly higher odds of being antigen positive (OR 20.4 for ICT, OR 31.8 for Og4C3 Ag >32 units, and OR 23.5 for any antigen) and antibody positive (OR 5.7 for Bm14 Ab and OR 9.5 for Wb123 Ab) than adult residents of non-hotspot areas. Compared to this reference adult group, residents of Ili’ili/Vaitogi/Futiga also had significantly higher odds of being positive for Og4C3 Ag of >32 units (OR 4.1) and Wb123 Ab (OR 2.3), but not for ICT (OR1.6) or Bm14 Ab (OR 1.0).

**Table 3:** Odds ratios for presence of serological markers for adult residents of hotspot villages compared to adult workers living in other villages (adjusted for age).

Presence of serological markers	Groups	Number tested	Number positive (%)	OddsRatio (OR)	*p* value
**ICT**	Residents of Fagali’i	38	7 (18.4)	20.4	**<0.001**
	Residents of Ili’ili/Vaitogi/Futiga	113	3 (2.7)	1.6	0.539
	Adult workers living in other villages	602	8 (1.3)	1	
**Og4C3 Ag >128**	Residents of Fagali’i	38	7 (18.4)	51.9	**<0.001**
	Residents of Ili’ili/Vaitogi/Futiga	112	4 (3.6)	5.2	**0.023**
	Adult workers living in other villages	598	4 (0.7)	1	
**Og4C3 Ag >32**	Residents of Fagali’i	38	8 (21.1)	31.8	**<0.001**
	Residents of Ili’ili/Vaitogi/Futiga	112	6 (5.4)	4.1	**0.016**
	Adult workers living in other villages	598	7 (1.2)	1	
**Antigen positive (ICT and/or Og4C3 Ag >32**	Residents of Fagali’i	38	9 (23.7)	23.5	**<0.001**
	Residents of Ili’ili/Vaitogi/Futiga	113	6 (5.3)	2.6	0.098
	Adult workers living in other villages	602	9 (1.5)	1	
**Bm14 Ab**	Residents of Fagali’i	38	17 (44.7)	5.7	**<0.001**
	Residents of Ili’ili/Vaitogi/Futiga	112	15 (13.4)	1.0	0.931
	Adult workers living in other villages	598	70 (11.7)	1	
**Wb123 Ab**	Residents of Fagali’i	38	20 (52.6)	9.5	**<0.001**
	Residents of Ili’ili/Vaitogi/Futiga	112	22 (19.6)	2.3	**0.014**
	Adult workers living in other villages	598	65 (10.9)	1	

## Discussion

Our results confirm that adult residents of the two suspected hotspot areas were significantly more likely to be seropositive for Og4C3 Ag and Wb123 Ab compared to adult residents of other villages in American Samoa. Residents of Fagali’i were also significantly more likely to be positive on ICT and seropositive for Bm14 Ab. We confirmed the presence of ongoing transmission in the suspected hotspot areas by identifying microfilaraemic residents, including a 9-year-old child. The results of this study therefore support the previous findings of suspected hotspots using a serum bank collected in 2010 for a leptospirosis study [19], and provide field evidence that WHO’s recommendations for integrating LF surveillance activities with other population-based surveys are potentially effective and feasible. However, the current study does not allow us to determine whether the hotspots represent areas of persistent transmission that were not successfully interrupted by MDA, or newly formed hotspots after MDA was completed.

Furthermore, our study confirmed ongoing transmission even though American Samoa passed TAS-1 of 6 to 7 year old children in 2011–2012 [17] and again passed TAS-2 in 2015 [18] (conducted a few months after this study). Our findings suggest that testing adults is a potentially effective surveillance strategy, particularly if performed in conjunction with TAS and used as baseline data. However, this strategy might require a sampling scheme quite different from the current WHO recommended sampling methods for TAS. In the post-MDA setting, when overall prevalence is very low and typically even lower in young children, testing adults might be more accurate for determining transmission status and more sensitive for identifying hotspots.

The age-adjusted estimates of the prevalence of all serological markers were higher in adult residents of Fagali’i compared to those who lived outside of hotspot villages. The seroprevalence of antigen and Wb123 Ab (but not Bm14 Ab) were higher in Ili’ili/Vaitogi/Futiga compared to residents of non-hotspot villages, but differences were not statistically significant, either because of the small sample size or the true absence of any difference. Although seroprevalence estimates for each group were standardised for age, the variations in age distribution between the groups could have made it more difficult to identify statistical differences.

Adult residents of both hotspots had significantly higher odds of being seropositive for Og4C3 Ag and Wb123 Ab than residents of other (non-hotspot) areas. Adult residents of Fagali’i also had higher odds of being seropositive for ICT and Bm14 Ab, compared to residents of other areas, but this was not found in Ili’ili/Vaitogi/Futiga. The reasons for the differences in the patterns of serological markers between the two hotspot areas are not clear, but could potentially be attributed to differences in intensity of transmission; or the timing of possible reintroduction or resurgence; or differences between persistent transmission from before MDA versus reintroduction or resurgence. The age-adjusted estimates of overall seroprevalence of all antigens and antibodies were significantly higher in Fagali’i compared to Ili’ili/Vaitogi/Futiga, suggesting higher transmission intensity in Fagali’i both recently and in the past. Previous studies in children have demonstrated the appearance of antigen and Wb123 Ab earlier in the course of infection than Bm14 Ab [27]. In Ili’Ili/Vaitogi/Futiga, the higher odds of being seropositive to antigen and Wb123 Ab (but not Bm14 Ab), together with ICT-positive 6–7 year olds in the local school in both TAS-1 and TAS-2, might reflect more recent reintroduction and/or resurgence compared to Fagali’i.

Our previous study found that recent migrants to American Samoa (mostly from Samoa) had significantly higher antigen and antibody prevalence [19], but this study did not find any significant difference in seroprevalence and number of years lived in American Samoa. A possible explanation is that more time has lapsed since MDA, and there is less difference in infection risk and/or the impact of MDA between long-term residents and recent migrants.

The strengths of our study include the large proportion of the population tested in the hotspot areas as well as the general population, and the wide range of age groups included. Our study population was highly stable and allowed accurate assessment of geographic variations in risk; >70% of each group had lived in American Samoa since the PacELF programme commenced in 2000. Our results should also be considered in light of the study’s limitations. Because of financial constraints and limited resources, the study was conducted using convenience sampling instead of probability-based sampling. Our reference group (adult workers who lived in other villages) were over 15 years of age but children were included in the residents of hotspot areas. Our previous study found that recent migrants who had not lived in American Samoa from the beginning of PacELF were more likely to be seropositive for antigen and antibody [19]. A lower proportion of our reference group (70.7%, 95% CI 66.8–74.3%) had lived in American Samoa since the beginning of PacELF compared to residents of Fagali’i (75.7%, 58.8–88.2%) and residents of Ili’ili/Vaitogi/Futiga (75.7%, 95% CI 66.6–83.3%). Our study only considered the place of residence, but infection could have occurred at work or elsewhere, especially when efficient day biters are present. Each of these three limitations could have weakened the associations between living in a hotspot and the presence of serological markers, but our study found statistically significant results despite the limitations. The age distributions of the hotspot residents and adult workers were significantly different to that of the general population, but the estimates of population seroprevalence were adjusted for age. Convenience sampling of hotspot residents and adult workers might have introduced bias towards people who have been diagnosed with LF, concerned that they might have the infection, or previously taken MDA. However, there was no evidence that any biases related to previous MDA or LF diagnosis were different between hotspot residents and adult workers.

Our findings raise a number of questions regarding current guidelines and targets used in LF elimination programmes, strategies for post-MDA surveillance, and transmission dynamics in the post-MDA setting. Firstly, our findings indicate that the current WHO recommended TAS has limitations in detecting ongoing transmission in the American Samoa setting. Our current study and previous studies in American Samoa [10,19] found evidence of ongoing transmission despite the territory passing TAS-1 in 2010/2011 and TAS-2 in 2015. In American Samoa, the antigen prevalence threshold used in school-based TAS of young children was not sensitive enough to detect ongoing low-level transmission. Future post-MDA surveillance strategies should consider including older children and adults, and/or determining thresholds that are more specific for different ecological settings [28]. In areas with highly efficient vectors (such as *Ae*. *polynesiensis* in American Samoa), LF transmission is likely to be more intense, and might therefore require different elimination targets to successfully interrupt transmission. Furthermore, TAS in American Samoa did not provide any indication of the high antigen prevalence in the Fagali’i hotspot even though >90% of elementary schools were included in the surveys. Our study identified heterogeneity in LF transmission at very small spatial scales, and concur with findings from diverse settings including Samoa [29], Haiti [30], Sri Lanka [31], and Zanzibar [32]. Our results also corroborate findings from Sri Lanka that TAS might not be sensitive enough for identifying small hotspots [31]. However, it is currently unclear whether these small residual foci of transmission will pose any significant risk of resurgence in the broader community, but the presence of microfilaraemic young children in these hotspots suggest that transmission is unlikely to disappear without intervention, particularly in areas with highly efficient vectors and strong environmental drivers of transmission.

Secondly, our findings raise concerns that in some settings, seven annual rounds of MDA might not be sufficient for interrupting transmission. Persistent transmission has been noted in Ghana [33] after up to 11 rounds of annual MDA, particularly in areas with high baseline Mf prevalence. In Zanzibar, ongoing transmission was detected six years after MDA, despite good coverage rates and Mf prevalence of <1% at sentinel sites after five rounds of MDA [32].

Thirdly, our results suggest that ICT might not be as sensitive as Og4C3 Ag or Wb123 Ab for detecting low-level transmission or resurgence, such as our hotspot in Ili’ili/Vaitogi/Futiga where Og4C3 Ag and Wb123 Ab provided warning signals, but ICT did not. In Mali, where MDA was conducted from 2002 to 2007, surveys in children in six formerly highly endemic villages found that ICT prevalence decreased from 53% pre-MDA to 0% (N = 120) after 6 rounds of MDA, and all adults tested in these villages were also antigen negative (N = 686) [34]. However, other longitudinal surveys in these villages using Og4C3 Ag and Wb123 Ab showed an increasing trend of antigen and antibody positivity in 6–7 year old children, from 0% in 2009 to 2.7% in 2011 and 4.5% in 2013, with one and three Mf positive children in 2009 and 2011 respectively [35]. Paradoxically, antigen prevalence by ICT in older children (>8 years) and adults decreased from 4.9% in 2009 to 2.8% in 2012. The results suggest that Og4C3 Ag and Wb123 Ab were more sensitive than ICT, and also raise the suggestion that in formerly highly endemic areas, adults might be immunologically protected while young children are susceptible and more rapidly infected.

Further research is being conducted in American Samoa to improve understanding of LF transmission in the post-MDA setting by conducting more representative sampling of all age groups in the general population, comparing the sensitivity of school-based versus community-based surveys, identifying risk factors for infection including the role of migrants, determining the spatial distribution and clustering of infected persons, and exploring the use of molecular xenomonitoring of mosquitoes.

## Supporting information

S1 Checklist STROBE checklist for cross-sectional studies. (DOC)

S1 Appendix Age distribution of study populations. (XLSX)

S2 Appendix Results of ICT, Og4C3 Ag, Wb123 Ab, and Bm14 Ab. (XLSX)
